# A randomized sequential cross‐over trial evaluating five purportedly ICP‐lowering drugs in idiopathic intracranial hypertension

**DOI:** 10.1111/head.14897

**Published:** 2025-01-24

**Authors:** James L. Mitchell, Hannah S. Lyons, Jessica K. Walker, Andreas Yiangou, Mark Thaller, Olivia Grech, Zerin Alimajstorovic, Georgios Tsermoulas, Kristian Brock, Susan P. Mollan, Alexandra J. Sinclair

**Affiliations:** ^1^ Translational Brain Science, Department of Metabolism and Systems Science, College of Medicine and Health University of Birmingham Birmingham UK; ^2^ Centre for Endocrinology, Diabetes and Metabolism Birmingham Health Partners Birmingham UK; ^3^ Department of Neurology Queen Elizabeth Hospital, University Hospitals Birmingham NHS Foundation Trust Birmingham UK; ^4^ Department of Military Rehabilitation Defense Medical Rehabilitation Centre Loughborough UK; ^5^ Department of Neurosurgery Queen Elizabeth Hospital, University Hospitals of Birmingham Birmingham UK; ^6^ Cancer Research Clinical Trials Unit University of Birmingham Birmingham UK; ^7^ Birmingham Neuro‐Ophthalmology Queen Elizabeth Hospital, University Hospitals Birmingham NHS Foundation Trust Birmingham UK

**Keywords:** adverse events, cerebrospinal fluid, cognition, idiopathic intracranial hypertension, intracranial pressure, therapeutics

## Abstract

**Objective:**

To gain initial insight into the efficacy to lower intracranial pressure (ICP), side effects, and effects on cognition of five drugs commonly used to treat idiopathic intracranial hypertension (IIH).

**Background:**

Limited clinical data exist for the treatment for IIH. Impaired cognition is recognized in IIH and can be exacerbated by medications.

**Methods:**

This human experimental medicine study was a secondary analysis that focused on an unblinded randomized, sequential, cross‐over extension of a previously completed randomized controlled trial. This study evaluated females with active IIH, recruited from University Hospital Birmingham, UK. Participants were treated, in randomized order, for 2 weeks with acetazolamide, amiloride, furosemide, spironolactone, and topiramate; assessment was at baseline and 2 weeks with a minimum 1‐week drug washout between drugs. The primary outcome was change in ICP at 2 weeks post‐drug administration. The cognitive evaluation was an exploratory study of the trial. ICP was recorded with telemetric, intraparenchymal ICP monitors (Raumedic, Hembrechts, Germany). Adverse events were recorded, and cognition was assessed utilizing the National Institutes of Health Toolbox Cognitive Battery.

**Results:**

Fourteen participants were recruited and evaluated by intention‐to‐treat analysis. Mean (standard deviation) body mass index was 37.3 (7.0) kg/m^2^ and ICP was 33.2 (7.1) cm cerebrospinal fluid (CSF) at baseline. ICP fell with four drugs (mean [standard error (SE)]), acetazolamide −3.3 (1.0) mmHg, *p* = 0.001, furosemide −3.0 (0.9) mmHg, *p* = 0.001, spironolactone −2.7 (0.9) mmHg, *p* = 0.003, and topiramate −2.3 (0.9) mmHg, *p* = 0.010. There was no significant difference between drugs. Side effects were common with acetazolamide (100%, 11/11) and topiramate (93%, 13/14). Baseline cognitive performance was impaired, *T*‐score (mean [SE]) 37.2 (2.6). After treatment, there was a further significant reduction in the fluid cognition domain (ability to process and integrate) with acetazolamide (mean *T*‐score [SE]), −5.0 (2.6), *p* = 0.057 and topiramate −4.1 (2.0), *p* = 0.061.

**Conclusions:**

Acetazolamide, furosemide, spironolactone, and topiramate marginally reduced ICP. While their effects were not significant, this study was not powered to detect a difference between drugs. Participants reported significant side effects with acetazolamide and topiramate including cognitive decline. Cognitive measures were impaired by acetazolamide and topiramate. Therapeutics with greater efficacy and a favorable side effect profile are an unmet need in the treatment of IIH.

## Plain Language Summary

This research studied how well five drugs treat idiopathic intracranial hypertension (IIH), which is a condition that causes high pressure in the brain. Most of the drugs slightly reduced pressure inside the head, but some caused side effects such as memory problems. There is still a need for better treatments with fewer side effects to treat patients with IIH.

**Abbreviations**
BD: twice a day
BMI: body mass index
HCG: human chorionic gonadotrophin
ICP: intracranial pressure
IIH: idiopathic intracranial hypertension
NIH: National Institutes of Health
RCT: randomized controlled trial
SD: standard deviation
SE: standard error

## INTRODUCTION

Idiopathic intracranial hypertension (IIH) is a systemic metabolic disease characterized by increased intracranial pressure (ICP). Chronic disabling headaches occur in the majority of patients with a risk of blindness.^1^, ^2^ There is no licensed treatment for IIH, which remains a fundamental unfulfilled clinical need for patients, as highlighted by a joint provider–patient group priority‐setting partnership.^3^ There is limited evidence for drug treatment in IIH, with only three randomized, placebo‐controlled trials having been conducted to date,^4^, ^5^, ^6^ with a sub‐cohort of participants from Mitchell et al. being analyzed in this study.^6^, ^7^, ^8^, ^9^, ^10^, ^11^, ^12^, ^13^ A 2015 Cochrane review concluded that there was not enough evidence to recommend or reject treatment with acetazolamide.^14^

Additionally, other drugs, including amiloride, furosemide, spironolactone, and topiramate, have been suggested as possible therapies for IIH and are used off label in clinical practice, but with low levels of evidence to date. Amiloride has been shown to reduce ICP in animal models via blockade of the Na+/H+ exchanger or Na+ channels,^15^, ^16^ and its use in IIH is reported in a case series.^17^ Furosemide is a loop diuretic that has been used to treat IIH;^18^ however, there is no direct evidence for reduction of ICP in IIH specifically, although there is evidence for reduction of ICP in other diseases in humans.^19^ Spironolactone is a K^+^‐sparing diuretic commonly used to treat heart failure and hypertension. The effect of spironolactone, along with chlorthalidone and sympathomimetic therapy, on orthostatic edema in IIH and matched control groups was assessed,^20^ but the study did not assess clinically relevant outcomes or ICP. Additionally, spironolactone has been observed to control IIH symptoms in a case series of patients with IIH and primary aldosteronism.^17^ Acetazolamide is a carbonic anhydrase inhibitor that is used in routine clinical care for patients with IIH.^21^ The IIH Treatment Trial evaluated visual outcomes with acetazolamide compared to placebo in conjunction with a low‐sodium weight‐reduction diet.^5^ This randomized controlled trial (RCT) demonstrated that acetazolamide, combined with the low‐sodium weight‐reduction diet, showed modest improvement in the visual field mean deviation compared to the placebo group. Topiramate also has carbonic anhydrase activity with the additional benefit of suppressing appetite. In an open label study, 40 patients with IIH were randomized to treatment with either topiramate or acetazolamide. Significant improvements were found in the visual fields for both groups, with no significant difference in efficacy between the groups.^22^

Treatment side effects are a concern in IIH and have been a reason to limit the dosage of acetazolamide.^4^, ^23^ Cognitive impairment is of particular concern in IIH, with patients commonly reporting symptoms of cognitive fogging,^24^, ^25^, ^26^, ^27^ which appears to be multifactorial and potentially reversible.^28^ Topiramate is known to have deleterious effects on cognition, affecting many domains, including verbal fluency, executive function, and working memory.^1^, ^29^, ^30^ Avoidance of medicines that adversely affect cognition would be preferable; however, no study has evaluated the effect on cognition of the most commonly used therapeutics in IIH.

The potential benefit of any medication needs to be balanced against the side effect profile. Therefore, the aim of this experimental medicine study was to gain insight into the ability of five drugs commonly prescribed for IIH to reduce ICP and assess the incidence of side effects and effects on cognition. We hypothesized that the administration of the commonly used drugs would reduce ICP and may impair cognition in IIH.

## METHODS

### Summary

This study evaluated 14 females with active IIH who had previously finished participating in a parallel‐arm RCT. This was an unblinded cross‐over extension for which they consented.^6^ They received sequential treatments, monitored by telemetric ICP catheters, with cognitive assessments using the National Institutes of Health (NIH) Toolbox. The focus was on ICP changes after 2‐week drug administration, alongside tracking adverse events and cognitive shifts.

### Trial design

This human experimental medicine study with a prospective, randomized, sequential, cross‐over design was conducted in females with active IIH. These individuals had finished participating in a parallel‐arm RCT and volunteered to participate in this unblinded cross‐over extension trial.^6^ Patients with a diagnosis of active IIH were identified and recruited from a single tertiary referral hospital (University Hospitals Birmingham NHS Foundation Trust). The trial was conducted according to the original protocol.

### Participants

Females aged 18–60 years who met the diagnostic criteria for definite IIH were recruited.^31^ All had normal brain imaging (aside from radiological signs of raised ICP), including magnetic resonance venography or computed tomography venography. All eligible patients had active IIH (optic nerve head swelling in at least one eye and ICP ≥ 25 centimetre of cerebrospinal fluid (cm CSF)). Eighteen patients were screened, with 14 enrolled. Those with significant co‐morbidities or previous surgery for IIH, including prior cerebrospinal fluid diversion procedures, were excluded. Those on medication that might influence ICP were advised to discontinue at least 1 month prior to enrollment. Patients who were pregnant or those planning pregnancy were excluded, and urine human chorionic gonadotrophin (HCG) was quantified at the first trial visit, along with renal function checked (blood urea and electrolytes). Detailed enrollment criteria are provided in the Table S1 in supporting information.

### Assessments

Following enrollment, a telemetric ICP catheter (Raumedic, Germany) was surgically implanted prior to the baseline visit (this approach to ICP monitoring was chosen by the patient advisory group and only offered to patients in whom it was also in line with clinical care decisions). The rationale for selecting the Raumedic P‐tel telemetric device to monitor ICP is its established accuracy and representativeness.^32^, ^33^ The Raumed P‐tel telemetric device has shown an excellent overall test–retest reliability (Pearson correlation 0.98, *p* < 0.001) and very good internal consistency reliability (Cronbach alpha 0.99).^34^ At baseline, medical history, examination (including blood pressure), and body mass index (BMI; calculated using the formula BMI = [weight (kg)/height (m)^2^]) were recorded. Papilledema was confirmed on a dilated slit lamp examination by a neuro‐ophthalmologist prior to surgical implantation of an ICP monitor as previously described.^6^

ICP was recorded using a transdermal telemetric (wireless) ICP monitoring system (Raumedic, Germany) at each drug baseline visit and after 2 weeks. ICP data were collected at a frequency of 5 Hz, and the mean ICP was calculated for each 30 min of continuous ICP monitoring in a validated standardized supine position, as previously described.^8^ Recordings were downloaded and analyzed in Dataview version 1.2 (Raumedic, Germany). ICP was recorded in mmHg (conversion factor to cmCSF was 1.36).

### Randomization and trial treatment

Participants were assigned treatments sequentially in a randomized cross‐over order using a simple computer‐generated randomization list. This was randomized and concealed from researchers by the University of Birmingham Clinical Trials Unit before administration to prevent selection bias. Treatment allocation was 1:1. The researcher was informed of treatment group allocation at the points of administration. The clinician evaluated eligibility, obtained informed consent, and enrolled the participants. Wash‐out between treatments was a minimum of 7 days (reflecting at least five and a half drug half‐lives as per the US Food and Drug Administration guidance of washout).^35^ Drug doses and titration are recorded in Table S2 in supporting information. Drug doses were reflective of typical clinical practice in IIH.^21^ Participants were dosed for 2 weeks (self‐administered at home), and compliance (pill counting) was evaluated on completion.

### Cognitive testing

Detailed evaluation of cognition was undertaken utilizing the NIH Toolbox Cognitive Battery (version 1.11).^36^, ^37^ This battery has a strong test–retest reliability for adults with an intraclass correlation coefficient ranging from 0.78 to 0.99 (good to excellent), with most values falling above 0.90.^36^ The battery consists of seven standardized testing paradigms measuring different cognitive constructs (Table S3 in supporting information). The battery consists of a standardized assessment covering a broad range of cognitive domains. It utilizes a computer adaptive testing paradigm, allowing assessments to be completed in 40 min. The battery has been validated in a US population across a large normative population (4859 individuals) and a broad array of acquired brain injuries.^37^ Test scores are expressed as fully corrected *t*‐scores (for which a score of 50 is population mean and ±10 is one standard deviation [SD] from mean), and individual patient scores are corrected for age, sex, educational attainment, and ethnicity.^38^ The summary scores describe crystalized and fluid components of cognition in line with the two‐component theory of intellectual development.^39^ In this paradigm, crystallized abilities are related to knowledge and experience, whereas fluid abilities reflect a person's capacity to process and integrate information and solve novel problems and are related to general intelligence. Tests were administered by a trained team member in a controlled, quiet environment under standard lighting conditions. Testing was performed at baseline and after 2 weeks of drug administration.

### Outcome measures

The primary outcome was a change in ICP at 2 weeks post‐drug administration. Secondary outcomes included adverse events and cognition.

### Adverse event reporting

Adverse events were recorded, along with drug compliance.

### Sample size calculation

As an exploratory study, no formal power calculation was undertaken. However, given the scarcity of trials in IIH and the absence of a clearly established minimal clinically important change for lumbar puncture pressure, it was recognized that this change could vary among individual patients. In the original study, the analysis aimed to achieve a significance level of at most *α* < 0.1 and a power of at least 80% using equal group sizes. Based on this criterion, the study determined that a total sample size of 14 patients was necessary.^6^ This calculation was grounded on an effect size of 6.5 cmCSF, along with a SD of 5.1 cmCSF, which represented the upper end of the range observed in a prior study.^40^ The study recruited patients who were available and willing to participate from the original study (recruitment was curtailed due to the COVID‐19 pandemic).

### Statistical analyses

All primary analyses (primary and secondary outcomes including safety outcomes) were evaluated by intention‐to‐treat analysis. Analysis was completed on received data, with every effort made to follow up participants to minimize potential for bias. Final analyses were conducted after the final visit of the final patient once the data had been cleaned and locked, then unblinded. No imputation of missing data was conducted.

Statistical analysis was performed in R v4.0.0 (R Foundation for Statistical Computing, Vienna, Austria). The hypothesis testing was conducted using a two‐tailed approach. Data were reported as means and SD (with median and interquartile range for non‐normal data), and standard error (SE) and 95% confidence intervals where appropriate. Hierarchical linear regression models were used to analyze the primary and secondary outcomes and to estimate differences between treatments. The response variable for each patient in each period was their change in outcome, that is, the end minus the start value. Each period in this cross‐over study was preceded by an appropriate washout period. In the hierarchical models, population‐level effects (also known as fixed effects) comprised the intercept and treatment. These terms captured the average outcome change over a treatment period irrespective of treatment and the average additional change in outcome associated with each treatment. Group‐level effects (also known as random effects) comprised patient‐level adjustments to the intercept. These terms captured the patient‐level tendencies for outcomes to improve or deteriorate and handled the serial correlation of outcomes within patient. A materially more complex model was not feasible, given the study sample size. The threshold for statistical significance was prespecified at 0.05. Data were parametric in Figure S2 in supporting information, and hence, a simple linear regression was used for analysis.

### Standard protocol approvals, registrations, and patient consents

This study was an exploratory study of the IIH Pressure trial, which was approved by the West Midlands–Solihull Research Ethics Committee (17/WM/0179) and all participants provided written informed consent according to Declaration of Helsinki principles. The IIH Pressure trial was registered with ISTCRN (12678718) and the first patient enrolled in June 2018.

## RESULTS

### Baseline characteristics

**FIGURE 1:**
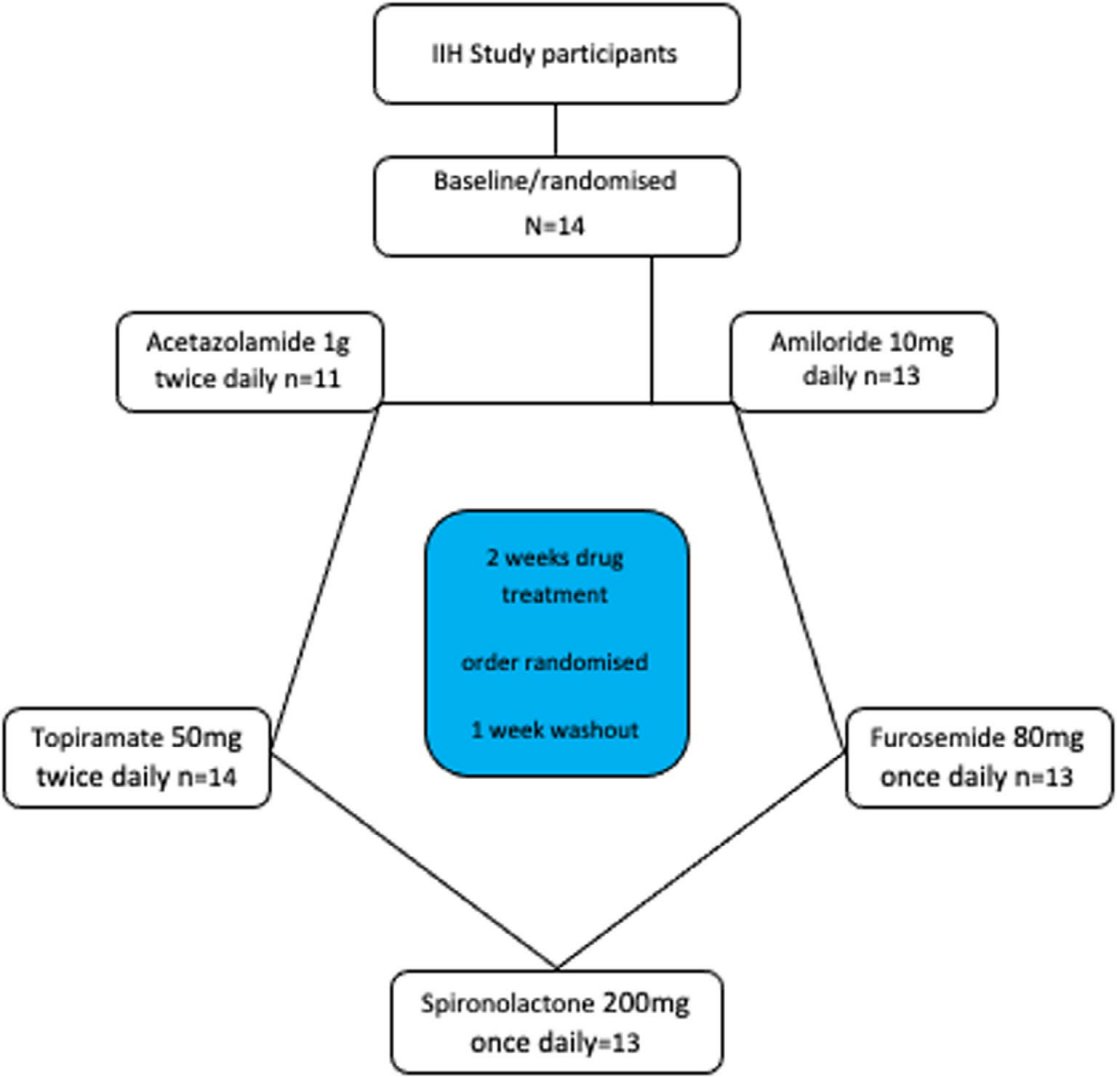
Consort diagram. Consort diagram describing the numbers and disposition of study participants. IIH, idiopathic intracranial hypertension. [Colour figure can be viewed at wileyonlinelibrary.com]

Fourteen female patients were recruited between June 2018 and March 2019 (Figure 1). At trial baseline, mean (SD) age was 28 (9) years, BMI 37.3 (7.0) kg/m^2^, and ICP 24.4 (5.2) mmHg (equivalent to 33.2 cmCSF; Table 1).

**TABLE 1:** Baseline characteristics.

Baseline characteristic	Mean (SD)
Number (*n*)	14
Age	28 (9)
BMI (kg/m^2^)	37.3 (7.0)
ICP (mmHg) supine	24.4 (5.2)
ICP (cm CSF) supine	33.2 (7.1)
Blood pressure (mmHg) systolic/diastolic	122/77 (11.2/5.0)

	Median (IQR)
Frisén grade at time of surgery (worst eye)	2 (1)

Abbreviations: BMI, body mass index; cmCSF, centimetre of cerebrospinal fluid; ICP, intracranial pressure; IQR, interquartile range; SD, standard deviation.

All participants had a normal renal function on blood test and all pregnancy tests (urine HCG) were negative.

### ICP change

**FIGURE 2:**
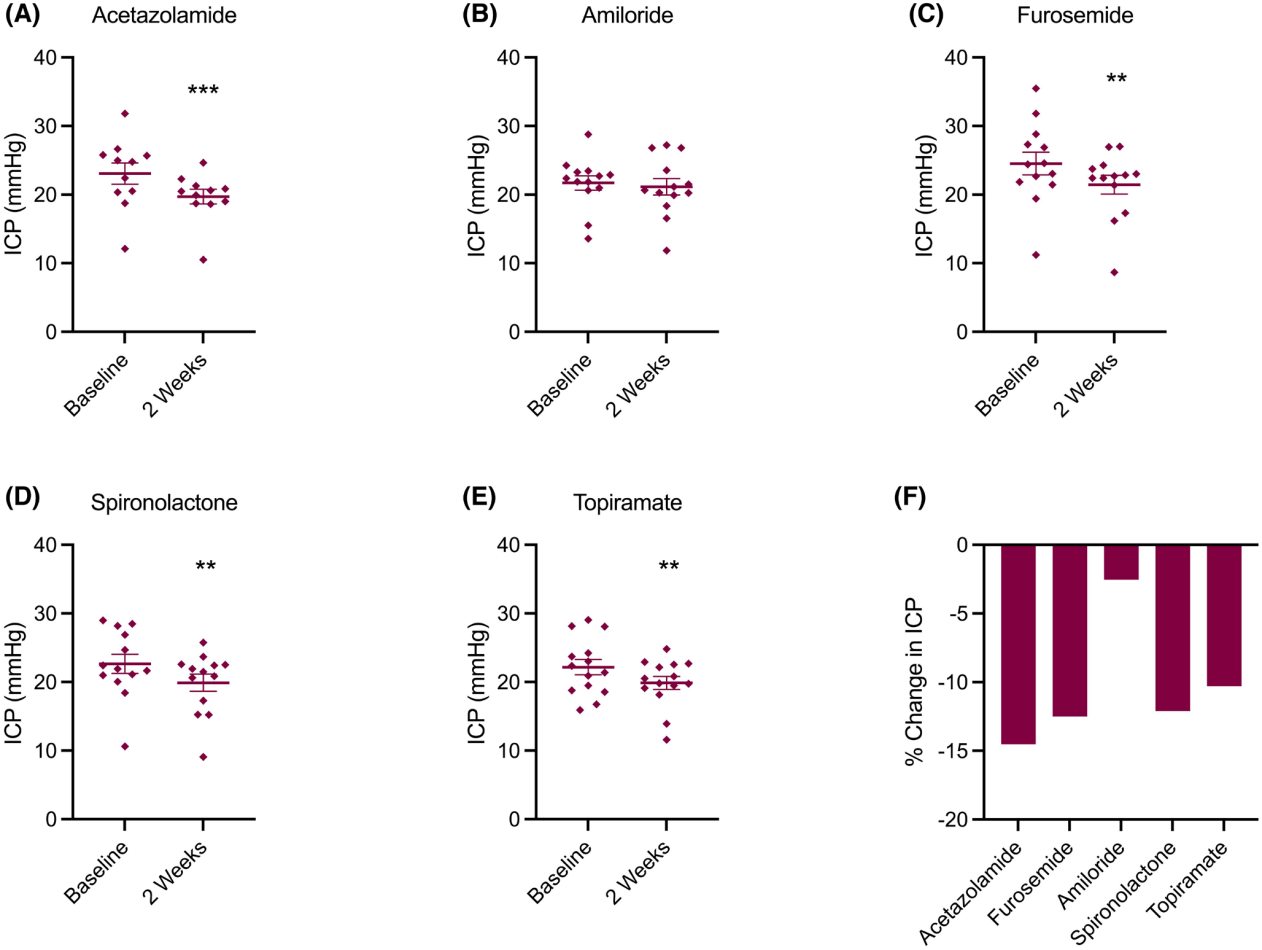
ICP change. (A–E) Mean ICP (mean [SEM]) at baseline and 2 weeks; (A) acetazolamide, (B) amiloride, (C) furosemide, (D) spironolactone, (E) topiramate. (F) Percentage change in ICP between baseline and week 2. **p < 0.01, ***p < 0.001 (significance level set at *p* < 0.05). Statistical values represent result from hierarchical linear regression model at the 2 week time point. ICP, intracranial pressure; SEM, standard error of the mean. [Colour figure can be viewed at wileyonlinelibrary.com]

Following 2 weeks of treatment, there was a fall in ICP recorded with all treatments apart from amiloride (Table 2, Figure 2). At 2 weeks, ICP change (mean [SD]) for acetazolamide was −3.3 (0.95) mmHg, *p* = 0.001; amiloride −0.5 (0.9) mmHg, *p* = 0.559; furosemide −3.0 (0.9) mmHg, *p* = 0.001; spironolactone −2.7 (0.9) mmHg, *p* = 0.003; and topiramate −2.3 (0.9) mmHg, *p* = 0.010 (Table 2, Figure 2). All telemeters functioned throughout the trial with no technical failure or indication of drift.^8^

**TABLE 2:** ICP change.

Treatment	*n*	Mean ICP pre‐drug (SEM) (mmHg)	Mean ICP post‐drug (SEM) (mmHg)	Mean change (SE) (mmHg)	Mean change (SE) (cmCSF)	Change (%)	*p*‐value
Acetazolamide	11	23.1 (1.5)	19.7 (1.1)	−3.3 (0.95)	−4.50 (1.3)	−14.5	0.001
Amiloride	13	21.7 (1.0)	21.2 (1.2)	−0.5 (0.88)	−0.70 (1.2)	−2.5	0.559
Furosemide	13	24.5 (1.7)	21.5 (1.4)	−3.0 (0.88)	−4.12 (1.2)	−12.5	0.001
Spironolactone	13	22.6 (1.4)	19.9 (1.3)	−2.7 (0.88)	−3.68 (1.2)	−12.1	0.003
Topiramate	14	22.2 (1.1)	19.9 (0.9)	−2.3 (0.85)	−3.11 (1.2)	−10.3	0.010

*Note*: Mean and SE.

Abbreviations: cmCSF, centimetre of cerebrospinal fluid; ICP, intracranial pressure; SE, standard error; SEM, standard error of the mean.

The magnitude of the treatment effect was compared for all treatment combinations. In this analysis, no treatment showed a statistically significant effect over any other (Table S4 in supporting information).

The magnitude of ICP reduction (% ICP change) was correlated with increasing baseline ICP. The higher the baseline ICP, the greater the reduction in ICP, *R* ^2^ = 0.12, *p* = 0.014 (Figure S2).

### Cognition

**FIGURE 3:**
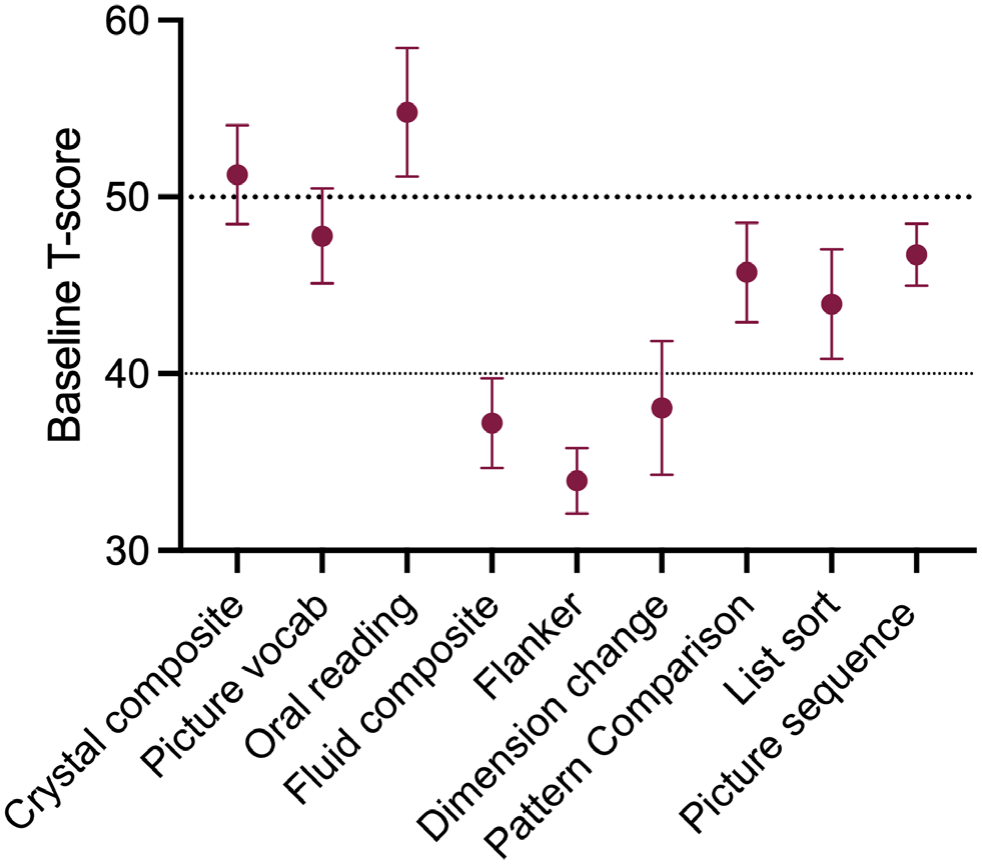
Baseline cognition. Baseline cognitive scores, mean (standard error of the mean), *t*‐scores fully corrected for age, sex, ethnicity, and educational attainment. [Colour figure can be viewed at wileyonlinelibrary.com]

At baseline, cognitive performance was reduced below a fully corrected *t*‐score of 50 for all fluid domains. Scores were more than one SD lower (fully corrected *t*‐score [SE]) for the fluid composite score 37.2 (2.6), flanker task 33.9 (1.9), and dimension change task 38.1 (3.8). Crystal composite, picture vocabulary, and oral reading scores were normal at baseline (Figure 3, Table S5 in supporting information).

**FIGURE 4:**
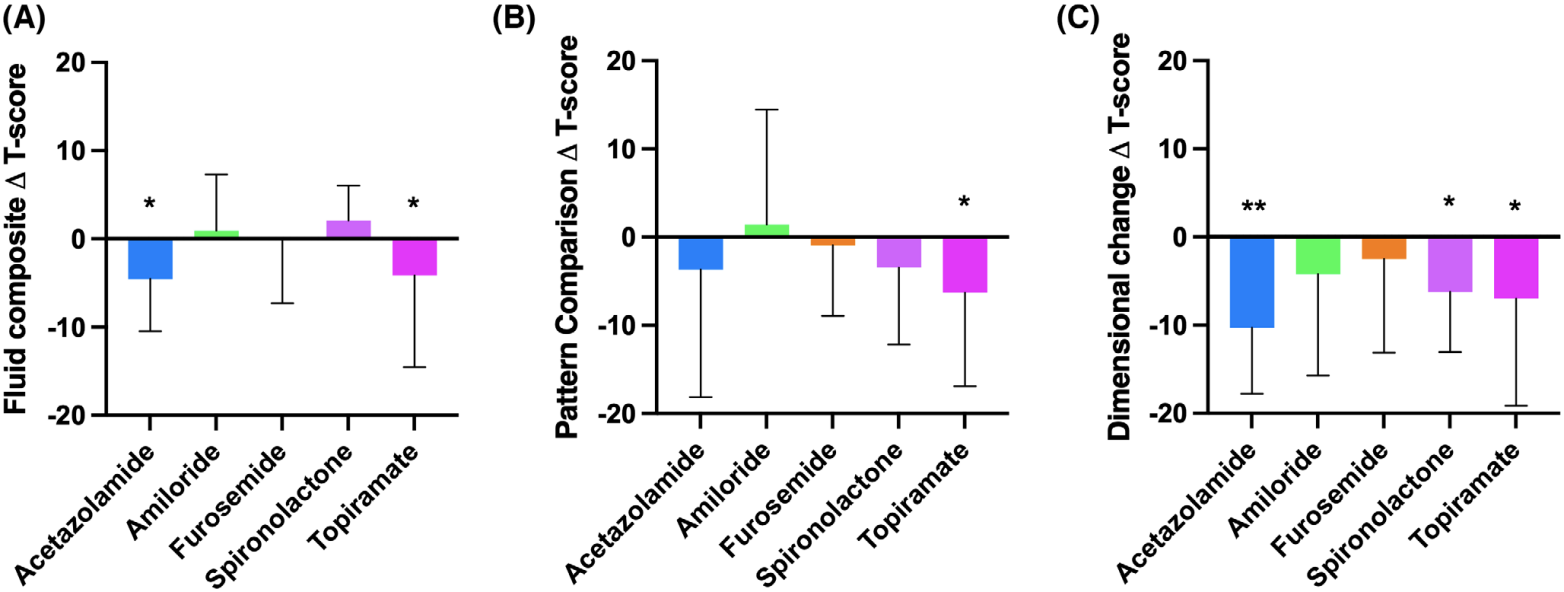
Cognitive change. (A–C) Change in fully corrected *t*‐score, mean (standard error of the mean), following 2 weeks of treatment: (A) fluid composite score, (B) pattern comparison task, (C) dimensional change task. *p < 0.05, **p < 0.01 (significance level set at *p* < 0.05). [Colour figure can be viewed at wileyonlinelibrary.com]

Within the fluid domain tasks, there were reductions in performance seen in the dimension change task with acetazolamide −10.3 (3.2), *p* = 0.002; spironolactone −6.2 (2.8), *p* = 0.030; and topiramate −7.0 (2.7), *p* = 0.012. Topiramate also caused a reduction in the pattern comparison task, −6.3 (2.9), *p* = 0.037. There was a non‐significant reduction in the fluid composite score associated with acetazolamide −5.0 (2.6), *p* = 0.057 and topiramate −4.1 (2.0), *p* = 0.061. There were no significant effects in the fluid domain for amiloride or furosemide (Figure 4, Table S6 in supporting information). There was no significant effect of any drug on the crystal composite, picture vocab, or oral reading recognition scores (Figure S1 in supporting information).

### Headache

There was no significant change in headache symptoms with the medications evaluated (Figure S3 in supporting information).

### Adverse events

There were no serious adverse events reported during the trial.

**FIGURE 5:**
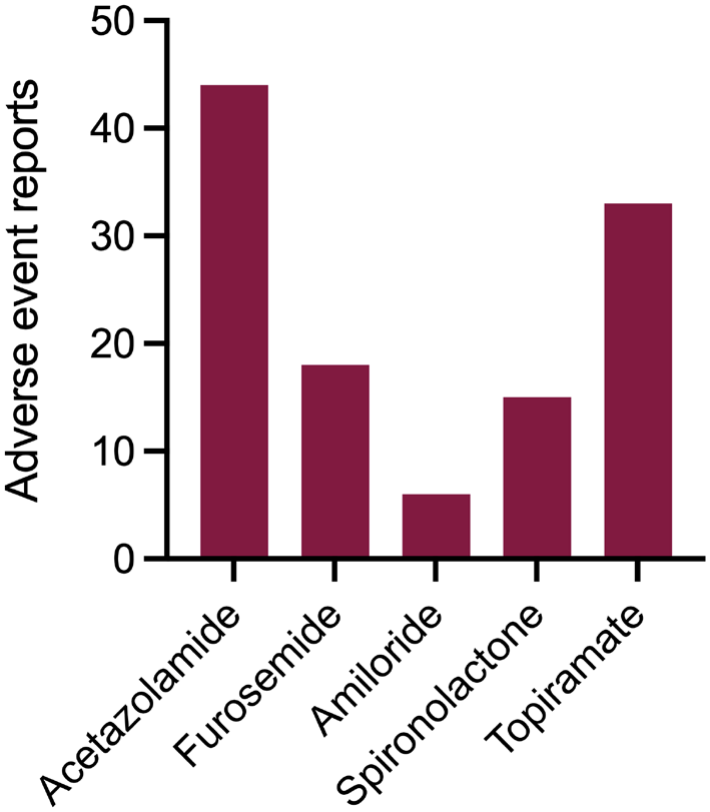
Adverse event reports. Number of adverse events reported for each drug. [Colour figure can be viewed at wileyonlinelibrary.com]

Drug‐related adverse events are reported in Table S7 in supporting information. Participants were asked to report drug‐related adverse events following 2 weeks of receiving each medication. The most commonly reported drug‐related adverse events were paresthesia (16 reports, 11 for acetazolamide) and nausea (13 reports). Cognitive disturbance was reported by 10 participants (71%) following topiramate but only 1 participant (9%) with acetazolamide. Participants taking acetazolamide also reported paresthesia (100%), dysgeusia (64%), and nausea (55%). Lethargy was reported by three participants on topiramate and five following acetazolamide. The drug with the least reported side effects was amiloride, and the drug with the most reported side effects was acetazolamide (Figure 5).

Some participants were unable to tolerate the maximum drug dosage (trial dosages reported in Table S8 in supporting information). Two participants had to reduce doses of acetazolamide, with one taking 500 mg twice a day (BD) and the other 250 mg BD for the final 7 days. One participant stopped taking spironolactone on day 10 and another participant stopped taking topiramate on day 4.

Of the 14 participants, not all took all five drugs (*n* = 11 acetazolamide, *n* = 13 amiloride, *n* = 13 furosemide, *n* = 13 spironolactone, *n* = 14 topiramate). Missing data were due to patient preference as some participants declined to take certain medications.

## DISCUSSION

This is a randomized sequential cross‐over trial to compare the effect of drugs commonly used in the treatment of IIH and to evaluate the effect on ICP, side effects, and cognition. This trial demonstrated that acetazolamide, furosemide, spironolactone, and topiramate all significantly reduced ICP, while amiloride had no significant effect. The range of ICP reduction with these drugs was between −3.1 and −4.5 cmCSF. Interestingly, the magnitude of ICP reduction correlated with increasing baseline ICP; thus, greater ICP reduction was observed in participants with higher starting ICP, which may have implications in determining when to use these drugs in clinical practice.

IIH is a chronic disease with intermittent relapses. Raised ICP (≥ 25cmCSF) is the hallmark of the disease, resulting in the common sequelae of headache and papilledema. The minimal clinically important change in ICP in those with IIH has not yet been fully determined.^41^ There have been five previous RCTs in IIH^4^, ^5^, ^6^, ^42^, ^43^ and one prospective cross‐over trial.^40^ Of these, four have utilized ICP as an outcome measure, with the magnitude of treatment effect between trial arms ranging from −5.6 cmCSF (exenatide vs. placebo),^6^ −5.9 cmCSF (acetazolamide and diet vs. placebo and diet),^5^ −6.0 cmCSF (bariatric surgery vs. Weight Watchers),^43^ and −6.2 cmCSF (low calorie diet vs. no diet).^40^ The efficacy in these studies is greater than that observed in this trial, but the treatment duration in this study was shorter (2 weeks). In the head‐to‐head comparison, acetazolamide, amiloride, furosemide, spironolactone, and topiramate did not have significantly greater effects than each other, although it should be noted that this study was not powered to detect a difference between drugs. We observed that the magnitude of ICP reduction correlated with increasing baseline ICP. Therefore, the higher the baseline ICP, the greater the reduction in ICP (*R* ^2^ = 0.12, *p* = 0.014). This could suggest that patients with more severe disease are more likely to experience reductions in ICP with drug therapy.

Cognitive fogging has been discussed by patients as a difficult symptom of their disease and this has been assessed in several small studies. Executive function has been demonstrated to be impaired, using a bespoke battery of cognitive tests, in IIH compared to age‐, sex‐, and BMI‐matched controls.^28^ They found that the cognitive impairment was reversible with decreased ICP, and that cognition was also influenced by headache severity, depression, and sleep apnea. The effects of drug treatment on cognition in IIH have not previously been formally evaluated. Both acetazolamide and topiramate have been known to cause adverse cognitive side effects in other diseases.^29^, ^44^

We demonstrated that at baseline, cognition was reduced in IIH with cognitive performance in the fluid domain more than one SD below expected, which is noted to be clinically significant.^45^ The fluid domain performance was particularly reduced in the flanker 33.9 (1.9) and dimension change paradigms 38.1 (3.8), which reflect attention and executive function. The remaining fluid domains were reduced but not below one SD.

There was no significant change in the cognitive tests with amiloride, furosemide, and spironolactone administration. There was a non‐significant reduction in the fluid composite score associated with acetazolamide and topiramate. Within the fluid domain, there were significant reductions in performance seen in the dimension change test with acetazolamide and topiramate, an assessment of executive function. Topiramate also caused a significant reduction in the pattern comparison score, which assesses processing speed. Hence topiramate and acetazolamide appeared to compound the existing deficits in the fluid domain. Interestingly, cognitive side effects were more commonly reported by participants with topiramate, which might in part reflect a nocebo effect due to inclusion of cognitive side effects in the patient information given during the trial. Participants demonstrated normal cognitive ability in the crystalized domains at baseline and there was no significant effect of any drug on crystalized cognitive scores, as would be expected.

This trial has several limitations. As an exploratory, sequential, randomized, cross‐over trial, we only intended to compare different drugs. The lack of a placebo group limits the interpretation of efficacy for each individual drug. However, inclusion of a matched placebo for each of the five drugs would have been implausible with this trial design. The inclusion of a placebo group would have been helpful for understanding the placebo response. Participants had undergone cognitive tests at baseline and following drug treatment; this repeated testing may have caused a learning effect that could have confounded the results. Learning effects with repeated testing are known to confound cognitive testing results in many studies of this nature. In our study, this was mitigated by incorporating variations in the cognitive testing paradigms, ensuring random allocation to drugs over time, and considering the repeat testing in our statistical analysis. In addition, it is known that the learning effect is dependent on the paradigm and cognitive domain evaluated; in this study, our results are in the executive function and processing speech domains, which are minimally affected by learning effects compared to working memory, for example, which can be very affected. But these were not the key measures of interest where we observed differences in this study. Previously, headache severity has been found to be adversely associated with cognitive performance in patients with IIH, although we did not explore the association between headache and cognitive performance due to the small group size. Regression to the mean is a possible explanation of the observed reduction in ICP; however, the design of the study with randomized sequence and the effect being seen in four of five drugs tested would make that less likely. We had standardized dose titration in this trial; this may have led to some patients being titrated more rapidly than would be done in clinical practice and this could have increased the adverse event reporting. Most clinicians gradually increase the dose of a drug to minimize side effects. This helps eliminate or reduce adverse events reporting. We acknowledge that a slower titration regimen may have reduced the reported side effects; nevertheless, this approach could possibly diminish the impact on ICP, given the extended duration required to reach the maximum tolerated effective dose. Exploration of the effect of titration on cognition would be a future interest to study.

We understand that the cohort was small; however, due to the constraints of trial design and the use of telemetric ICP monitors, further recruits were not possible. For practicalities, there was a shorter dosing duration (2 weeks), and this limited the ability to escalate to higher drug doses. Consequently, our results cannot be extrapolated to other drug doses. The short duration of dosing also limits the interpretation of the results over a longer period to realize the full benefit in terms of clinical measures. We note, however, that ideally, drugs to lower ICP in IIH would show initial efficacy within the first 2 weeks. Drugs that took longer than this to affect ICP are less clinically useful. As an exploratory trial, ours was not powered to detect differences between drugs. Not everyone received the same dose due to the pragmatic nature of dosing and an individual's inability to tolerate certain doses.

It is known that ICP and clinical features of IIH can gradually improve over time;^43^, ^46^ therefore, the ICPs were not matched at the start of each drug dosing. There was no significant difference between the starting ICPs for the different drugs.

While there was no control group, the cognitive comparisons were made to the NIH Toolbox Cognition Battery normative population (*n* = 1038),^38^ which is validated in the United States as opposed to the UK. Although educational and cultural effects would be expected to be seen in the crystalized scores, this was not observed in this experimental study. A key unique strength of the study was the use of telemetric monitors to measure ICP and the ability to measure ICP for prolonged periods over several weeks without further invasive procedures, such as multiple lumbar punctures. The intracranial telemetric monitors were safe, and all monitors functioned throughout the duration of this trial.

## CONCLUSION

In this exploratory randomized, sequential, cross‐over trial with drugs evaluated over 2 weeks, ICP was reduced by the administration of acetazolamide, furosemide, spironolactone, and topiramate, although whether this represents a clinically meaningful change is unknown. We observed that cognition (attention and executive function) was impaired at baseline. Acetazolamide and topiramate both worsened the existing cognitive impairment in IIH, as well as having the most predominate adverse event profile. We observed that these medications were more effective at ICP reduction in those with highest ICP. These potential benefits and risks need to be balanced when evaluating the appropriateness of prescribing these unlicensed drugs in IIH. An effective, well tolerated licensed treatment remains an unfulfilled clinical need for people with IIH.

### AUTHOR CONTRIBUTIONS

Conception and design: JLM, SPM, AJS, GT. Acquisition of data: JLM, HSL, AY, MT, ZA. Analysis and interpretation of data: JLM, HSL, JKW, OG, KB, SPM, AJS. Drafting manuscript: JLM, HSL, AY, MT, OG, ZA, KB, SPM, AJS. Revising it for intellectual content: JLM, HSL, JKW, AY, MT, OG, ZA, SPM, AJS, GT. Final approval of the completed manuscript: all authors.

### CLINICAL TRIALS REGISTRATION NUMBER

The trial was registered with ISTCRN, no: 12678718. Submitted to the registry June 19, 2017.

## FUNDING INFORMATION

This trial was funded by Enterprising Birmingham, University of Birmingham, UK, from August 1, 2016. Further funding was sought and from August 1, 2019 an investigator‐led grant was obtained from Invex Therapeutics. James L. Mitchell was funded by the Ministry of Defence for the duration of the trial. Andreas Yiangou was funded by an Association of British Neurologists and Guarantors of the Brain Fellowship. Olivia Grech was funded by Brain Research UK. Alexandra J. Sinclair was funded by a National Institute for Health Research (NIHR) clinician scientist fellowship (NIHR‐CS‐011‐028) and the Medical Research Council, UK (MR/K015184/1) for the duration of the trial. Alexandra J. Sinclair is funded by a Sir Jules Thorn Award for Biomedical Science. The views expressed are those of the authors and not necessarily those of the UK National Health Service, MoD, NIHR, nor the UK department of Health and Social Care. Role of Funder/Sponsor: The MoD, NIHR, MRC, and Invex Therapeutics had no role in the design or conduct of the trial; no role in collection, management, or interpretation of the data; writing of the manuscript; and no role in the decision to submit the manuscript for publication.

## CONFLICT OF INTEREST STATEMENT

**James L. Mitchell** was funded by the UK Ministry of Defense for the duration of the trial. **Olivia Grech** reports scientific consultancy fees from Invex Therapeutics (2020). **Andreas Yiangou** reports receiving speaker fees from Teva, UK, outside the submitted work. **Kristian Brock** works for AstraZeneca; and owns shares in GSK. **Susan P. Mollan** reports consultancy fees (Invex Therapeutics), advisory board fees (Invex therapeutics; Gensight), speaker fees (Heidelberg engineering; Chugai‐Roche Ltd.; Allergan; Santen; Chiesi; and Santhera), and travel (Abbie Vie). **Alexandra J. Sinclair** reports personal fees from Invex Therapeutics in her role as director with stock holdings, during the conduct of the trial (since 28.06.2019); others from Allergan, Novartis, Cheisi, and Amgen outside the submitted work. **Hannah S. Lyons, Jessica K. Walker, Andreas Yiangou, Mark Thaller Zerin Alimajstorovic**, and **Georgios Tsermoulas** declare no conflicts of interest.

## Supporting information


**Data S1.**.

**Figure S1.**.

**Figure S2.**.

**Figure S3.**.

## Data Availability

Individual participant data, after anonymization, will be made available, along with the study protocol, statistical analysis plan, and consent forms. Reasonable requests will provide data beginning 12 months and ending 3 years after publication of this article to researchers whose proposed use of the data is approved by the original study investigators. Proposals should be made to the corresponding author, and requesters will need to sign a data access agreement.
