# Supplementary material for: A randomized sequential cross‐over trial evaluating five purportedly ICP‐lowering drugs in idiopathic intracranial hypertension
Source: Headache. 2025 Jan 24;65(2):258–68. doi: 10.1111/head.14897 (PMC11794974; doi:10.1111/head.14897)
Supplement: Supplementary file 1 — Data S1.. [file HEAD-65-258-s002.docx]

**A randomised sequential cross-over trial evaluating five purportedly ICP lowering drugs in idiopathic intracranial hypertension.**

**Supplementary tables:**

| **Inclusion Criteria** |
| --- |
| Female patients with IIH, aged between 18 and 60 years, diagnosed according to the modified Dandy criteria who have active disease (papilloedema [Frisen grade ≥ 1], significantly raised ICP > 25cmCSF) and no evidence of venous sinus thrombosis (magnetic resonance imaging (MRI) or computerized tomography (CT) imaging and venography as noted at diagnosis). |
| Able to give informed consent. |
| **Exclusion Criteria** |
| Age less than 18 or older than 60 years. |
| Pregnant or trying to conceive. |
| Significant co-morbidity, such that in the opinion of the investigator, it would not be in the participant’s best interest to participate in the trial. |
| Addison’s or Cushing’s disease. |
| CSF shunt/stent or optic nerve sheath fenestration. |
| Currently using GLP-1 agonist or DPP-4 inhibitor. |
| Surgical contra-indication. |
| Concomitant therapy with acetazolamide, topiramate, or diuretics (this can be discontinued 1 month prior to enrolment). |
| Inability to give informed consent, e.g., due to cognitive impairment. |

**Supplementary Table 1** **Inclusion and exclusion criteria.** CT; computerised tomography, CSF; cerebrospinal fluid, DPP-4; dipeptidyl peptidase 4, GLP-1; glucagon-like peptide-1, IIH; idiopathic intracranial hypertension, ICP; intracranial pressure, MRI; magnetic resonance imaging.

| **Drug** | **Starting dose** | **Final dose** |
| --- | --- | --- |
| **Acetazolamide** | 500mg BD | 1G BD |
| **Amiloride** | 10mg OD | 10mg OD |
| **Furosemide** | 40mg OD | 80mg OD |
| **Spironolactone** | 100mg OD | 200mg OD |
| **Topiramate** | 25mg BD 4 days, 25/50mg 3 days | 50mg BD |

**Supplementary Table 2** **Drug doses.** BD, twice daily; OD, once daily.

| **Test** | **Cognitive domains** |
| --- | --- |
| **Flanker Inhibitory Control and Attention Test** | Attention and executive function |
| **Picture Sequence Memory Test** | Episodic memory |
| **List Sorting Working Memory Test** | Working memory |
| **Picture Vocabulary Test** | Language |
| **Oral Reading Recognition Test** | Language |
| **Dimensional Change Card Sort Test** | Executive function |
| **Pattern Comparison Processing Speed Test** | Processing speed |

**Supplementary Table 3 Cognitive testing domains.**

| **Treatment comparisons** | **ICP mean change difference (SE) (mmHg)** | **p=** |
| --- | --- | --- |
| **Acetazolamide vs Amiloride** | -2.8 (1.2) | 0.128 |
| **Acetazolamide vs Furosemide** | -0.3 (1.2) | 0.999 |
| **Acetazolamide vs Spironolactone** | -0.6 (1.2) | 0.985 |
| **Acetazolamide vs Topiramate** | -1.0 (1.1) | 0.897 |
| **Amiloride vs Furosemide** | 2.5 (1.1) | 0.172 |
| **Amiloride vs Spironolactone** | 2.2 (1.1) | 0.286 |
| **Amiloride vs Topiramate** | 1.8 (1.1) | 0.483 |
| **Furosemide vs Spironolactone** | -0.3 (1.1) | 0.998 |
| **Furosemide vs Topiramate** | -0.7 (1.1) | 0.959 |
| **Spironolactone vs Topiramate** | -0.4 (1.1) | 0.995 |

**Supplementary Table 4** **Drug effect on ICP head-to-head comparison.**

| **Cognitive test** | **T-score mean (SE)** |
| --- | --- |
| **Crystal composite** | 51.3 (2.8) |
| Picture vocab | 47.8 (2.7) |
| Oral reading | 54.8 (14.1) |
| **Fluid composite** | 37.2 (2.6) |
| Flanker | 33.9 (1.9) |
| Dimension change | 38.1 (3.8) |
| Pattern Comparison | 45.7 (2.8) |
| List sort | 43.9 (3.1) |
| Picture sequence | 46.7 (1.8) |

**Supplementary Table 5** **Baseline cognitive scores.**

| **Test** | **Treatment** | **T-score change mean (SE)** | **p=** |
| --- | --- | --- | --- |
| **Crystal composite** | Acetazolamide | -0.6 (2.1) | 0.777 |
|  | Amiloride | 0.9 (1.8) | 0.605 |
|  | Furosemide | -0.1 (1.8) | 0.968 |
|  | Spironolactone | 2.1 (1.9) | 0.267 |
|  | Topiramate | -0.2 (1.8) | 0.905 |
| **Picture vocab** | Acetazolamide | 2.9 (2.7) | 0.286 |
|  | Amiloride | 1.0 (2.3) | 0.662 |
|  | Furosemide | -0.4 (2.3) | 0.851 |
|  | Spironolactone | 1.6 (2.4) | 0.496 |
|  | Topiramate | 0.6 (2.3) | 0.778 |
| **Oral reading recognition** | Acetazolamide | -4.3 (2.7) | 0.120 |
|  | Amiloride | 0.8 (2.3) | 0.734 |
|  | Furosemide | 0.4 (2.3) | 0.853 |
|  | Spironolactone | 1.9 (2.4) | 0.443 |
|  | Topiramate | -0.9 (2.3) | 0.688 |
| **Fluid composite** | Acetazolamide | -5.0 (2.6) | 0.057 |
|  | Amiloride | -0.9 (2.2) | 0.695 |
|  | Furosemide | -0.3 (2.2) | 0.896 |
|  | Spironolactone | -1.4 (2.3) | 0.542 |
|  | Topiramate | -4.1 (2.2) | 0.061 |
| **Flanker** | Acetazolamide | -1.4 (2.4) | 0.557 |
|  | Amiloride | 2.0 (2.0) | 0.328 |
|  | Furosemide | 1.8 (2.0) | 0.382 |
|  | Spironolactone | -0.5 (2.1) | 0.802 |
|  | Topiramate | 3.0 (2.0) | 0.145 |
| **Dimension change** | Acetazolamide | -10.3 (3.2) | 0.002 |
|  | Amiloride | -4.2 (2.7) | 0.125 |
|  | Furosemide | -2.5 (2.7) | 0.359 |
|  | Spironolactone | -6.2 (2.8) | 0.030 |
|  | Topiramate | -7.0 (2.7) | 0.012 |
| **Pattern comparison** | Acetazolamide | -3.8 (3.5) | 0.280 |
|  | Amiloride | 1.4 (2.9) | 0.630 |
|  | Furosemide | -0.9 (2.9) | 0.754 |
|  | Spironolactone | -3.4 (3.1) | 0.265 |
|  | Topiramate | -6.3 (2.9) | 0.037 |
| **List sort** | Acetazolamide | 1.6 (2.4) | 0.507 |
|  | Amiloride | 1.8 (2.0) | 0.381 |
|  | Furosemide | 2.1 (2.0) | 0.294 |
|  | Spironolactone | 1.4 (2.1) | 0.512 |
|  | Topiramate | -2.9 (2.0) | 0.153 |
| **Picture sequence** | Acetazolamide | -2.9 (4.4) | 0.516 |
|  | Amiloride | -3.9 (3.7) | 0.299 |
|  | Furosemide | -1.6 (3.7) | 0.677 |
|  | Spironolactone | 4.1 (3.9) | 0.299 |
|  | Topiramate | -0.4 (3.7) | 0.924 |

**Supplementary Table 6** **Change in cognitive test scores following each treatment.**

| **Symptom** | **Acetazolamide**  **(n=11)** | **Amiloride**  **(n=13)** | **Furosemide**  **(n=13)** | **Spironolactone**  **(n=13)** | **Topiramate**  **(n=14)** |
| --- | --- | --- | --- | --- | --- |
| **Mood disturbance** | 1 | 0 | 0 | 0 | 4 |
| **Lethargy** | 5 | 0 | 0 | 2 | 3 |
| **Cognitive fog/memory** | 1 | 0 | 0 | 0 | 10 |
| **Paraesthesia** | 11 | 0 | 0 | 1 | 4 |
| **Visual disturbance** | 0 | 1 | 0 | 0 | 1 |
| **Coordination/balance** | 1 | 0 | 0 | 0 | 3 |
| **Palpitation** | 0 | 0 | 0 | 0 | 1 |
| **Nausea** | 6 | 0 | 4 | 1 | 2 |
| **GI upset** | 5 | 0 | 0 | 1 | 1 |
| **Taste disturbance** | 7 | 0 | 0 | 1 | 0 |
| **Diuresis** | 0 | 1 | 8 | 2 | 0 |
| **Menstrual disturbance** | 1 | 0 | 0 | 0 | 0 |
| **Dizziness** | 2 | 1 | 1 | 1 | 1 |
| **Muscle cramp** | 1 | 0 | 1 | 1 | 0 |
| **Headache** | 0 | 0 | 0 | 1 | 1 |
| **Thirst/dry mouth** | 0 | 2 | 0 | 1 | 1 |
| **Skin** | 0 | 0 | 0 | 2 | 0 |
| **Feel faint** | 0 | 0 | 2 | 1 | 0 |
| **Abdominal pain** | 0 | 1 | 1 | 0 | 0 |
| **Shortness of breath** | 3 | 0 | 0 | 0 | 0 |
| **Dry eyes** | 0 | 0 | 0 | 0 | 0 |
| **Sleep disturbance** | 0 | 0 | 0 | 0 | 1 |
| **Ankle swelling** | 0 | 0 | 1 | 0 | 0 |
| **Total** | **44** | **6** | **18** | **15** | **33** |

**Supplementary Table 7 Drug adverse events.**

| **Drug** | **Drug dose as per protocol (n=)** | **Reduced dose (n=)** |
| --- | --- | --- |
| **Acetazolamide** | 9 | 2 |
| **Amiloride** | 13 | 0 |
| **Furosemide** | 13 | 0 |
| **Spironolactone** | 12 | 1 |
| **Topiramate** | 13 | 1 |

**Supplementary Table 8 Drug dosing summary.**
