# Supplementary figures and images for: A randomized sequential cross‐over trial evaluating five purportedly ICP‐lowering drugs in idiopathic intracranial hypertension
Source: Headache. 2025 Jan 24;65(2):258–68. doi: 10.1111/head.14897 (PMC11794974; doi:10.1111/head.14897)

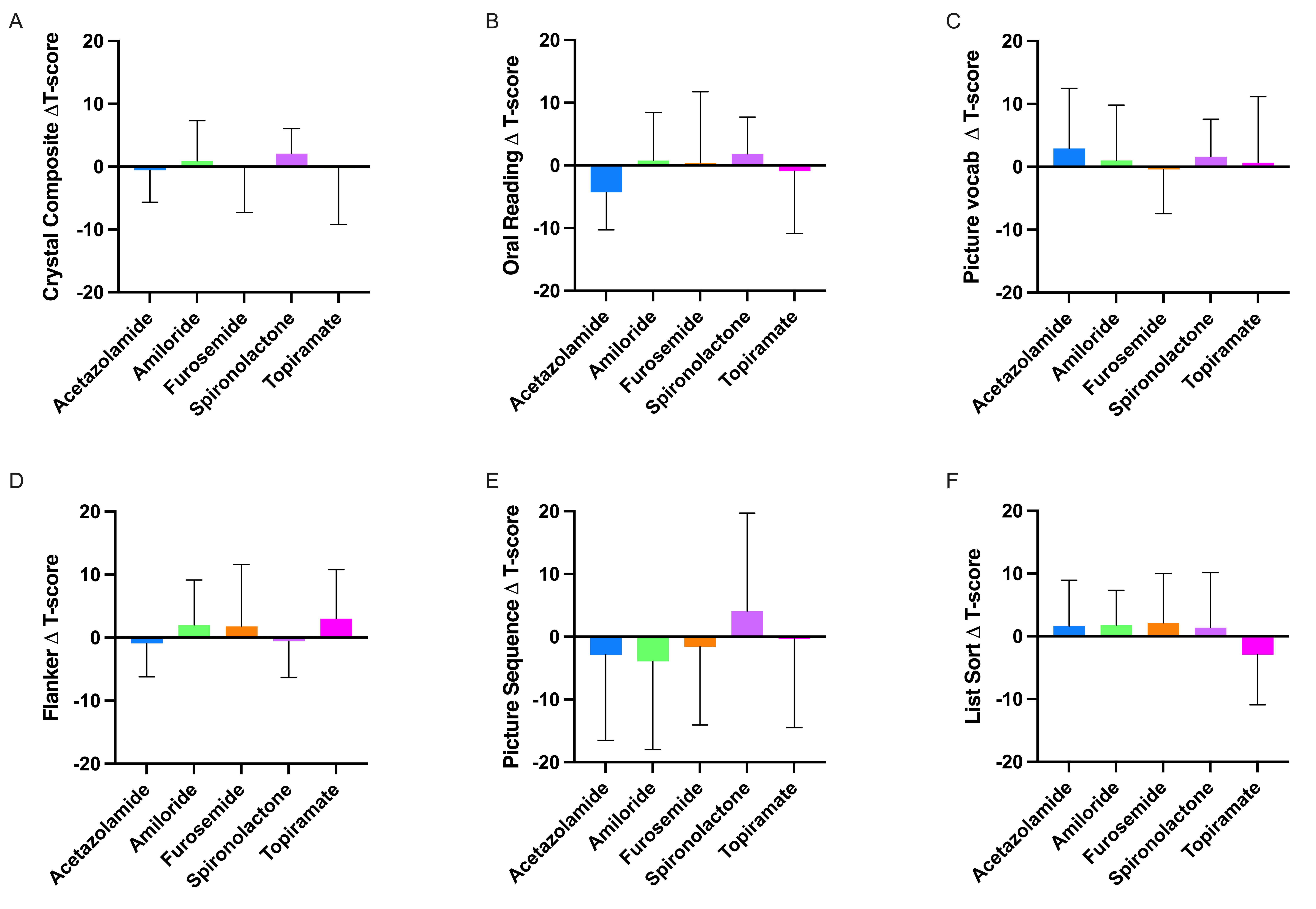

Supplement: Supplementary file 2 — Figure S1.. [file HEAD-65-258-s001.jpg]

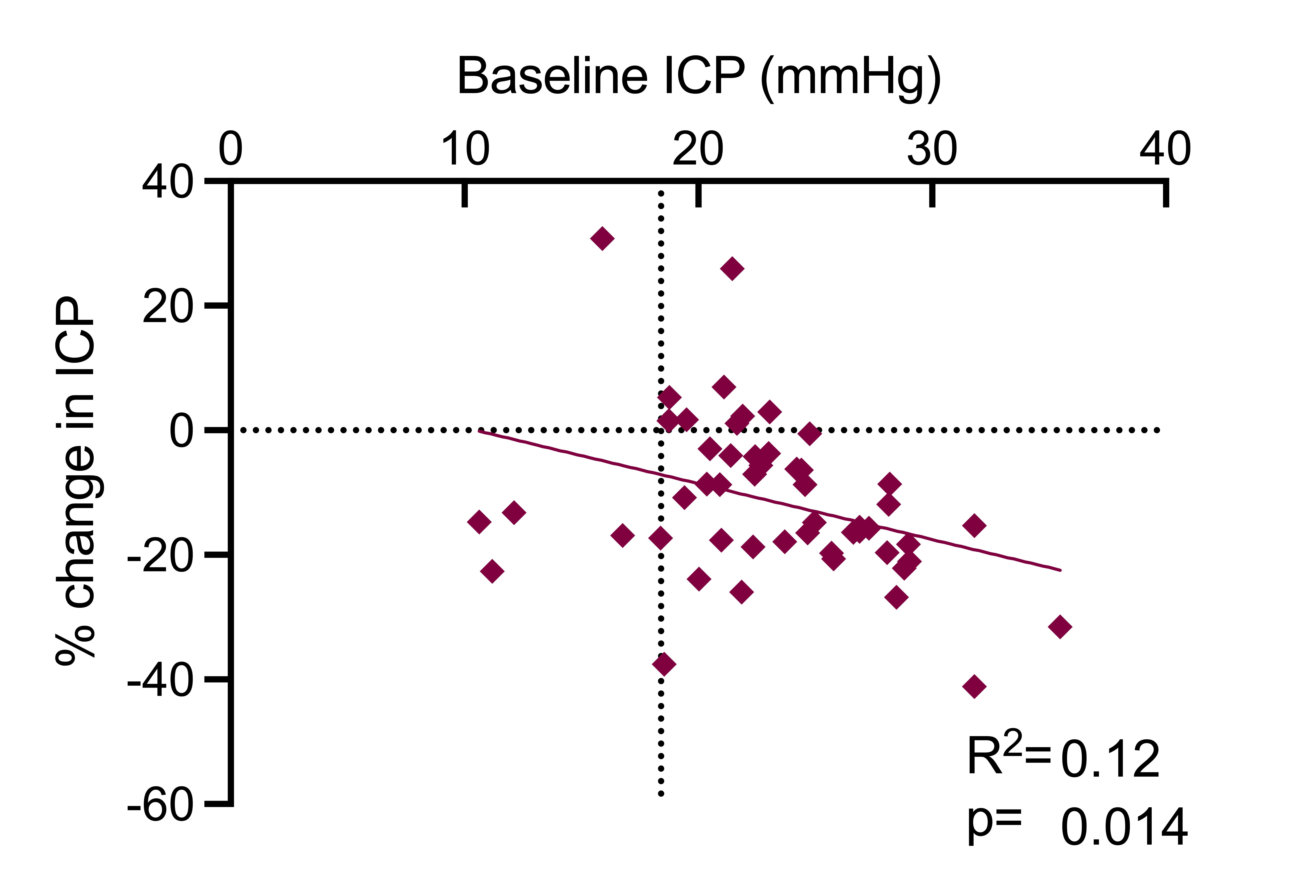

Supplement: Supplementary file 3 — Figure S2.. [file HEAD-65-258-s004.jpg]

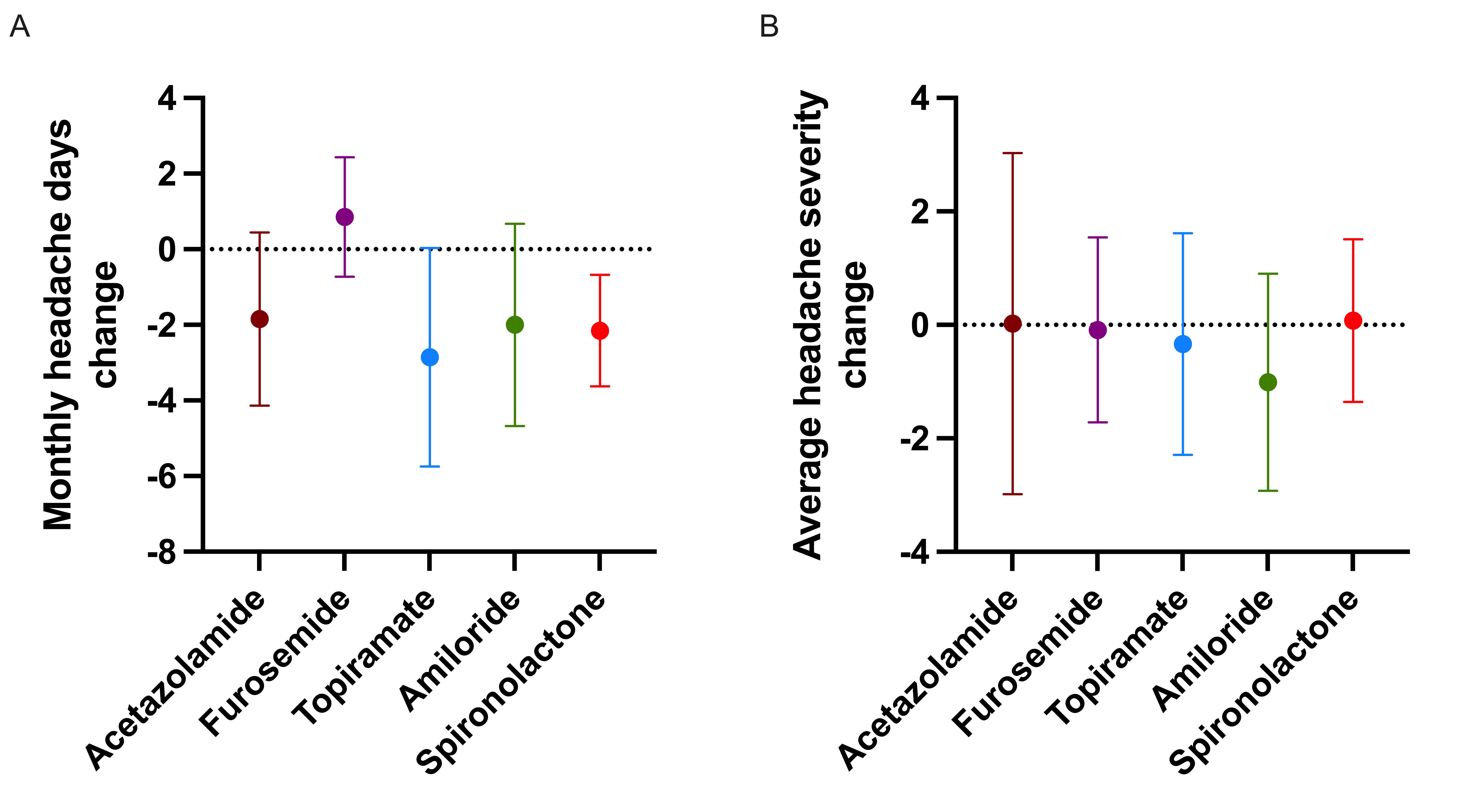

Supplement: Supplementary file 4 — Figure S3.. [file HEAD-65-258-s003.jpg]
